# Supplementary material for: The sociomotor role and its meaning in traditional sporting games: Socio-affective rejection in question
Source: PLoS One. 2025 Dec 5;20(12):e0336908. doi: 10.1371/journal.pone.0336908 (PMC12680248; doi:10.1371/journal.pone.0336908)
Supplement: S1 File — (DOCX) [file pone.0336908.s001.docx]

Inclusivity in global research

PLOS’ policy on inclusivity in global research aims to improve transparency in the reporting of research performed outside of researchers’ own country or community and ensures that PLOS publications reporting global research adhere to high standards for research ethics and authorship. Authors of relevant research articles may be asked to complete the questionnaire below, which outlines ethical, cultural, and scientific considerations specific to inclusivity in global research. This questionnaire may be requested when researchers have travelled to a different country to conduct research, if research uses samples collected in another country, research with Indigenous populations or their lands, or if research is on cultural artefacts. Researchers travelling to another country solely to use laboratory equipment will not normally be required to complete the questionnaire. However, the questionnaire can be requested at the journal’s discretion for any submission – if you have been requested to complete this questionnaire by the PLOS journal you submitted to, please do so.

Please complete the questionnaire below and include this as a Supporting Information file with your manuscript. Note that if your paper is accepted for publication, this checklist will be published with your article in the supporting information files. Please ensure that you reference the checklist in the main body of your manuscript. We suggest adding a subsection ‘Inclusivity in global research’ to your Methods section and adding the following sentence: “Additional information regarding the ethical, cultural, and scientific considerations specific to inclusivity in global research is included in the Supporting Information (SX Checklist)”

The questions have been designed to be applicable to a wide range of study types, and there are subsections for both human subjects research and non-human subjects research. If any of the questions are not relevant to your research please mark them as “N/A” as appropriate.

**Ethical considerations, permits and authorship**

*This section is applicable to all research types.*

Provide details as to who granted permissions and/or consent for the study to take place in the Methods section of your manuscript. This should include the names of **all** ethics boards, governmental organizations, community leaders or other bodies that provided approval for the study. If individuals provided approval refer to these people by their role or title but do not list their name(s).

Reported on page number: The study received approval Ethics Committee for Clinical Research of the Catalan Sports Council (reference: 09/CEIGGC/2020). This information can be found in the method section, under participants, on pages 7 and 8 of the manuscript.

If there were any deviations from the study protocol after approval was obtained please provide details of these changes in the Methods section of your manuscript.
Did this study involve local collaborators that are residents of the country where the research was conducted or members of the community studied? If you do not have any authors from said communities, please provide an explanation for this below.
Everyone listed as an author should meet PLOS’ criteria for authorship and all individuals who meet these criteria should be included in the author byline, rather than the acknowledgements. For further information please see the journal’s Authorship Policy.

All researchers listed as authors of this study are members of the Research Group on Motor Action (GIAM) at the National Institute of Physical Education of Catalonia (INEFC), which includes researchers from different countries. In this particular case, the research team is composed of investigators from Tunisia and Spain. The lead author is from Tunisia, and the remaining researchers are residents of the country where the study was conducted.
Although the study was conducted with students from INEFC as the sample, **no students were included as authors** of the article.

Reported on page number: Following approval, the study was conducted in full accordance with the approved protocol, with no deviations. This information can be found in the method section, under participants, on pages 7 and 8 of the manuscript.

**Human subjects research (e.g. health research, medical research, cross-cultural psychology)**

Did you obtain written informed consent from a representative of the local community or region before the research took place? How did you establish who speaks for the community? Details of written informed consent obtained from study participants should be reported separately in the Methods section of your manuscript.

All participants provided written informed consent prior to participation in the study.

How did members of the local community provide input on the aims of the research investigation, its methodology, and its anticipated outcome(s)?

A university session was conducted in which the research question, objectives, and study protocol were presented and discussed in detail. Students actively contributed by sharing their perspectives and constructive feedback. A dedicated follow-up session was organised to evaluate their comprehension of the study’s aims and to collect specific suggestions for refinement. This participatory process helped ensure that both the methodology and the expected outcomes were coherently aligned with the educational realities and professional aspirations of the student community..

When engaging with the local community, how did you ensure that the informed consent documents and other materials could be understood by local stakeholders?

An informed consent document was designed using language appropriate for the population participating in the study. To ensure its comprehensibility, a group of 40 students read the document and provided feedback on its content. These comments were reviewed by the research team, who then implemented the necessary modifications.

Will the findings of the research be made available in an understandable format to stakeholders in the community where the study was conducted (e.g. via a presentation, summary report, copies of publications, etc.)? Please provide details of how this will be achieved.

he students involved in this research project are enrolled in the double degree in Physiotherapy and Physical Activity and Sport, as well as in the Bachelor’s degree in Physical Activity and Sport Sciences at the National Institute of Physical Education of Catalonia (INEFC), affiliated with the University of Lleida, Catalonia, Spain. Their participation was conceived as a formative experience, providing them with first-hand exposure to the design and implementation of a scientific study — from the formulation of the research question and objectives to the development of the study protocol. All research materials and consent forms were written in clear, accessible academic language, ensuring full comprehension by the participants.

Following the intervention, the study findings are presented during a dedicated classroom session. This meeting includes an open discussion aimed at promoting knowledge transfer and contextualising the results within students’ future professional practice. Through this process, participants are encouraged to reflect on the practical relevance and ethical implications of the research in their own educational and occupational settings.

**Non-human subjects research using specimens/ animals collected as part of the study, or those housed in archival collections. Examples include archaeology, paleontology, botany and zoology.**

Did the permission you obtained from a local authority to perform the study include an agreement on access to outputs and benefit sharing? This may include procedures to enable fair distribution of the benefits and resources arising from the research performed. Please include any details of Prior Informed Consent and Benefit Sharing Agreements obtained. These may be required by field-specific regulations, for example the Convention on Biological Diversity (CBD) and the associated Nagoya Protocol.

N/A.

If the material used in your study was imported, please A) provide the year it was imported and B) indicate whether permits were obtained to import/export the materials used, C) provide details of any permits obtained. If this information is not available, please indicate this.

N/A

If you used archival specimens, please state how the material used in your study was acquired by the institute it is held in and provide details of any permits obtained for the original excavations/ sample collection. If this information is not available, please indicate this.

N/A

How was the potential cultural significance of the materials collected in your study to local communities considered in your research design? Were Indigenous peoples and/or local researchers and institutions involved with archaeological excavations / collection of specimens? If so, please provide a description of their involvement.

N/A.

If your manuscript includes photographs of human remains please indicate whether authors obtained permission from descendants or affiliated cultural communities to do so.

N/A.
